# Parke, Davis & Co.

**Published:** 1882-01-20

**Authors:** 


MESSRS. PARKE, DAVIS & CO.—We acknowledge the receipt, in due time, of the beautiful card of the above staunch and excellent house of Detroit, extending to the managing editor of THE RECORD the compliments of the season. We warmly appreciate the token, and reciprocate the kind wishes of our friends so elegantly and courteously tendered.

